# Curvature Dynamics of PEGDA Asymmetric Networks via Frontal Photopolymerization: Effect of Chain Length and Optical Attenuation

**DOI:** 10.1021/acs.macromol.5c02783

**Published:** 2026-02-12

**Authors:** Muhammad Ghifari Ridwan, Huseyin Mirac Dizman, Isobel Bentley, Alessandra Vitale, João T. Cabral

**Affiliations:** † Department of Chemical Engineering, 4615 Imperial College London, London SW7 2AZ, U.K.; ‡ Department of Applied Science and Technology, 19032 Politecnico di Torino, Torino 10125, Italy

## Abstract

We investigate how the oligomer molecular mass, chain length, and optical attenuation affect both polymerization kinetics and the spatiotemporal response of materials patterned via frontal photopolymerization (FPP). We employ model poly­(ethylene glycol) diacrylate (PEGDA) oligomers of different chain lengths and investigate their FPP kinetics and response following solvent development, focusing on the emergence and evolution of the material curvature. We find that longer precursors yield a lower dose (or time) threshold for solidification, effectively benefiting from an “early start,” while the front velocity remains unchanged with chain length; by contrast, photoinitiator concentration leads to a nonmonotonic impact on kinetics due to the combined effects on rate and optical attenuation, which we collapse on a master curve. FPP networks can exhibit nonmonotonic, spontaneous curvature fluctuations, from flat or convex, to concave, and back to convex, that we show to depend on PEGDA chain length and describe by a minimal evaporation–diffusion model. These findings demonstrate how the interplay between molecular structure, soft mechanics, and solvent transport can be harnessed to program the response of asymmetric polymer networks.

## Graphical abstract

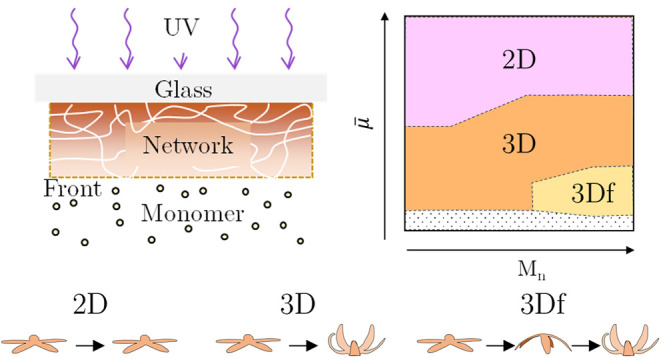

## Introduction

Concepts for 4D soft materials, systems that change shape or function over time with or without external stimuli, have developed rapidly over the past decade in polymer science.^1–4^ By contrast with conventional static polymers or network structures, 4D materials are programmed during fabrication to evolve predictably under environmental changes such as temperature,^5^ moisture,^6^ pH,^7^ light,^8^ or solvent swelling/deswelling.^9^ These transformations are driven by a symmetry-breaking property, for instance, an asymmetry in material architecture or physicochemistry, that results in autonomous, reversible, and stimulus-responsive behavior. Emergent phenomena in connected functional units have been demonstrated in a range contexts, where interactions lead to complex and often unexpected response.^10–12^ Such approaches unlock a range of possibilities for nonplanar material assembly and origami, where complex 3D shapes or motion arise from controlled deformation of initially flat or simple geometries.^5,13,14^

One powerful strategy for achieving such transformations in polymer networks is by engineering internal gradients throughout the material, such as variations in cross-link density, stiffness, swelling capacity, or residual stresses. These gradients induce nonuniform deformation upon stimulation, resulting in bending,^9^ twisting,^15,16^ folding,^9,14^ or buckling.^17^ Unlike mechanical actuators or multimaterial composites, such materials can encode “instructions” for shape change at the molecular level during synthesis. As a result, gradient-based 4D materials offer a scalable and elegant route to construct shape-shifting structures, ranging from soft robotics,^18^ sensors,^19^ and bioinspired devices.^20^

Frontal photopolymerization (FPP) is a versatile approach for creating asymmetric polymeric networks with exceptional control of the conversion both along the direction of illumination (*z*) and within the plane (*xy*), by employing a photomask or modulated illumination.^21–23^ In FPP, light initiates a planar polymerization front that travels through a liquid monomer or prepolymer, under conditions of strong light attenuation and limited mass and thermal diffusion conditions, converting it into polymer network.^24^ This process inherently generates spatial gradients in polymer conversion, leading to variations in cross-linking density and stiffness along the front’s propagation direction.^22^ Material gradients arise from differences in exposure time, heat generation, system chemical composition, radical diffusion, and polymerization kinetics. Unlike layer-by-layer methods, FPP enables single-step fabrication of morphologically dynamic materials with built-in structural heterogeneity.^9,13^

The cross-sectional profile of the traveling waves of network formation can be effectively tuned by adjusting formulation parameters and front propagation conditions,^23,25,26^ and under a range of practically relevant conditions, profiles are shape-invariant and exhibit predictive propagation kinetics, yielding well-defined asymmetric networks of prescribed dimensions. When exposed to an external stimulus, such as solvent swelling/deswelling, these networks undergo predictable deformation into complex nonplanar shapes.^5,9,13,27,28^ This curvature-inducing behavior is central to many natural systems, from plant movement to tissue morphogenesis,^29–31^ and is increasingly being replicated in synthetic materials.^32^

In this paper, we investigate the effect of the chain length of the precursor oligomer, employed as a “photoresist,” on the resulting asymmetric properties and stimuli-responsive behavior of 4D FPP networks. Our hypothesis is that the mass of a model difunctional oligomer can be an effective design parameter of gradient networks with prescribed mesh size and thus soft mechanics and transport properties.^34^ Understanding how chain length affects not only FPP kinetics but also the distribution of internal stresses and curvature evolution during solvent swelling and drying is essential for designing materials with a predictable 4D behavior. We select a difunctional acrylate oligomer, poly­(ethylene glycol) diacrylate or PEGDA, as a model system, as it can form networks and hydrogels with a range of practical biomedical, sensing, and separation applications. We examine the role of PEGDA chain length, optical attenuation, and photoinitiator stoichiometry in FPP kinetics and 4D response of asymmetric networks following solvent development. We demonstrate that oligomer chain length provides an effective lever to not only tune FPP propagation but also to selectively control the spatiotemporal response and nonplanar assembly of otherwise identical asymmetric FPP networks. We then introduce a minimal model that combines FPP kinetics with evaporation–diffusion of the solvent that can capture our experimental observations of emerging curvature and curvature fluctuations and thus provide a framework for the design of 4D materials based on structurally encoded gradients.

## Experimental Section

To fabricate asymmetric polymer networks with different molecular mesh sizes, poly­(ethylene glycol) diacrylate (PEGDA) with three different number-average molecular masses (*M*_*n*_) of 250, 575, and 700 g mol^–1^ (Sigma-Aldrich, references 475629, 437441, 455008) and photoinitiator (PI) phenylbis­(2,4,6-trimethylbenzoyl)­phosphine oxide (Irgacure-819, BASF, 30128871) were used. PI/PEGDA ratios ranging from 0.0005 to 0.07 w/w (defined as mass of PI over mass of PEGDA) were investigated (defined as mass of PI over mass of PEGDA). Batches of 15 g of PEGDA precursor and PI were mixed using a magnetic stirrer at 750 rpm for 1 h and subsequently wrapped with aluminum foil and stored at 4 ^o^C to prevent inadvertent polymerization. Ethanol (VWR Chemicals 99.97%, code 20821.321) was used for development (by solvent immersion) without further purification.

A collimated UV light source (Omnicure S1500, equipped with a 365 nm filter) was employed, and a VItec RS-365 digital radiometer (Spectroline) was employed to calibrate and measure irradiance, generally fixed between 2 and 2.5 mW/cm^2^. The PEGDA/PI mixture (∼0.625 mL) was placed between two microscope glass slides (1 × 3 in., Fisherbrand 1238–3118) separated by 1–3 mm spacers. Photomasks were designed using AutoCAD (2024) and printed onto the acetate film using a laser printer at 600 dpi resolution, and placed atop the upper glass slide. After exposure, the printed polymeric network (∼0.02 mL) was pad dried, and its thickness was measured using a digital caliper (Mitutoyo, model PK-0505CPX). The sample was then developed by immersion in ethanol (typically 10 mL). The resulting structures were imaged using a BASLER acA640-750uc camera fitted with a 0.5–1X Edmund Optics lens (87535).

## Results and Discussion

We first consider the kinetics of network formation by frontal photopolymerization (FPP), depicted in Figure 1a. Upon UV light irradiation, a polymerization front propagates through the resin, forming a well-defined boundary that separates the unreacted precursor from the cross-linked network. After removal of excess liquid PEGDA and pad drying, the position of the front *z*_f_ along the illuminated axis (*z*) defines the network thickness for a given irradiation dose *d*.^21,24^ The resulting network has an asymmetric cross-section described by an order parameter ϕ­(*z*), defined between 0 and 1, corresponding to neat precursor and full conversion, respectively. The illuminated surface exhibits a greater ϕ, which decreases toward the network–liquid interface, where ϕ ≡ ϕ_c_, a system-specific threshold value for network formation. The sigmoidal ϕ­(*z*) profile, defined by an interfacial width *w*, propagates during photopolymerization as a planar wave, with a shape-invariant profile in many practically relevant conditions.^25,35^

**Figure 1:**
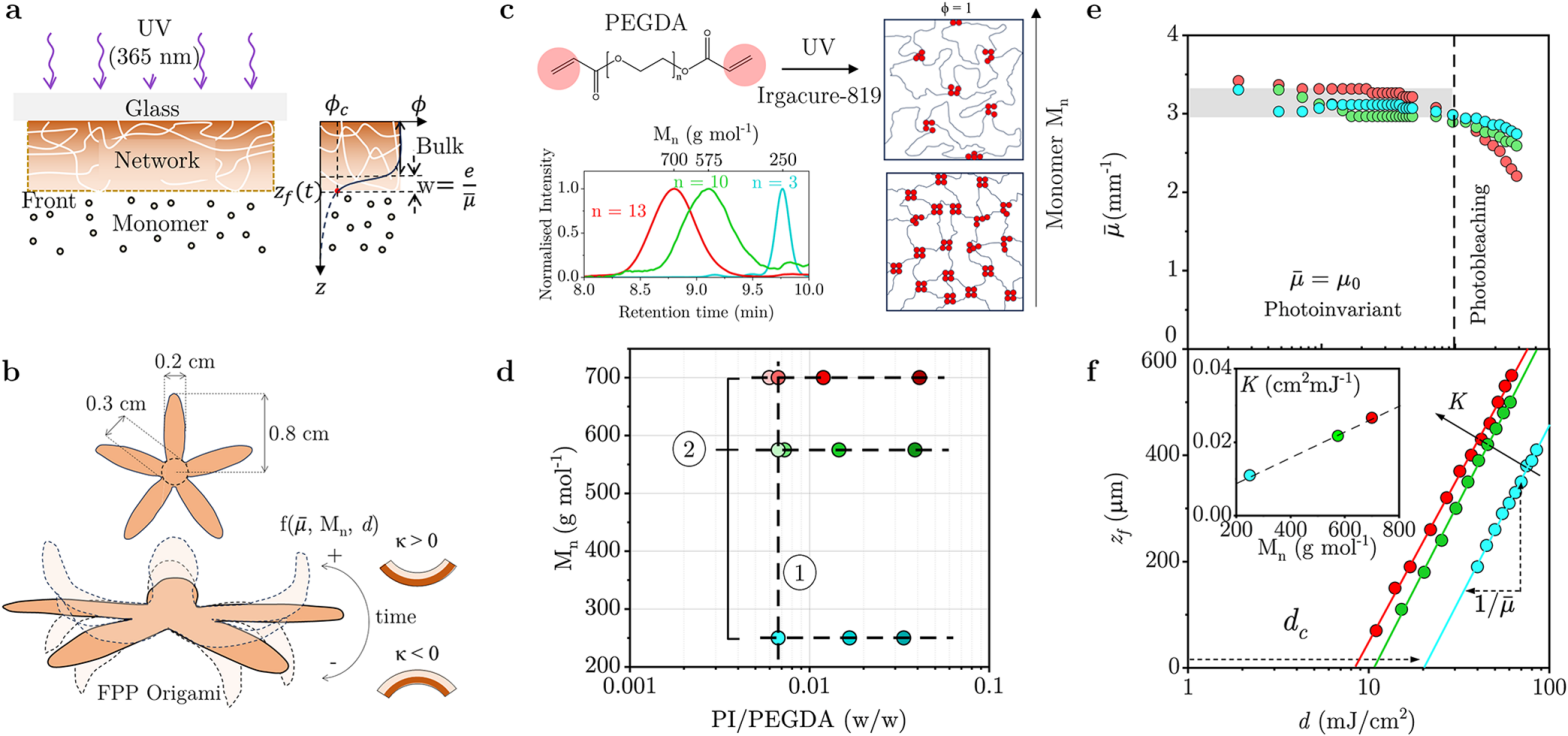
(a) FPP schematic depicting the frontal network formation, accompanied by a sharp conversion profile ϕ­(*z*), with front position *z*_f_ corresponding to the intersection with a critical conversion value ϕ = ϕ_c_, yielding an interfacial width *w* governed by optical attenuation coefficient μ̅. (b) Nonplanar material design (illustrated with a “starfish” pattern) and schematic of dynamic response governed by oligomer molecular mass (*M*_*n*_), optical attenuation μ̅, and irradiation dose *d*. Curvature κ is defined in terms of the network asymmetry, κ > 0 if it curves toward less cross-linked interface, and κ < 0 if toward the illuminated surface. (c) Chemical structure of the PEGDA oligomer and size distribution obtained by GPC for PEGDA with *M*_*n*_ of 250 (blue), 575 (green), and 700 (red) g mol^–1^ corresponding to 3, 10, and 13 ethylene glycol (EG) repeat units. Schematic of network formation for low and high *M*_*n*_ at full conversion ϕ ≡ 1. (d) Experimental map of PI/PEGDA (w/w) ratio at varying *M*_*n*_, defining representative isopleths ① and ②. The gradient of color intensity corresponds to the PI fraction. (e) Evolution of optical attenuation coefficient μ̅ with irradiance dose *d*, where the vertical dashed line indicates conditions up to which the process is effectively photoinvariant (μ̅ ≈ μ_0_). Data for *M*_*n*_ 700 g mol^–1^ have been previously reported^33^ and are included here for comparison. (f) Logarithmic growth of front position, *z*_f_, with irradiation dose, *d*, with a proportionality constant of 1/μ̅ and an *x*-axis intersection at the critical irradiation dose *d*_c_, as described by eq 2. The inset shows that the effective FPP conversion constant *K* increases linearly with PEGDA *M*_*n*_.

Figure 1b depicts a starfish-shaped pattern (*xy*) and possible spatiotemporal responses for asymmetric networks, namely, the emergence of curvature κ toward the illuminated surface (κ < 0) or toward the network–liquid interface (κ > 0), which we hypothesize to be a function of the optical properties, precursor molecular mass, and irradiance. This “starfish” illustrative geometry enables a visualization of network response to molecular and FPP parameters and development. We select PEGDA of three different number-average molecular masses (*M*_*n*_) 250, 575, and 700 g mol^–1^ which are liquid at room temperature, with ethylene glycol repeating units *n* = 3, 10, and 13, respectively (Figure 1c). Higher *M*_*n*_ (≥1000 g mol^–1^) correspond to PEGDA that are solid at room temperature and thus not suited as liquid FPP photoresists. An illustration of fully converted (ϕ = 1) networks architecture of high and low precursor *M*_*n*_ is also provided.

### FPP Coarse-Grained Model

To interpret our experimental results, we employ a well established coarse-grained analytical model describing the kinetics of FPP.^21–26,36,37^ The simplest, minimal model is based on a typical first-order reaction equation of normalized monomer-to-polymer conversion ϕ, and a differential Beer–Lambert law to capture the directional light attenuation of the medium during photoconversion. As the directional solidification progresses, conversion may induce changes in light attenuation, which is modeled as a composition-average optical attenuation coefficient, μ̅(*z*, *t*) = μ_0_(1 – ϕ­(*z*, *t*)) + μ_∞_ϕ­(*z*, *t*), where μ_0_ is the coefficient of the neat resin and μ_∞_ is the coefficient of the fully converted material. While in general μ̅ evolves over the course of the reaction, in the case of photoinvariant systems (or regimes), μ̅ ≈ μ_0_. An analytical expression for the normalized conversion profile along the solidification direction (*z*-axis) is then obtained

1 ϕ(z,d)=1−exp[−Kdexp(−μ̅z)]

By defining the normalized critical monomer-to-polymer conversion ϕ_c_ as the value of ϕ at the front position *z*_f_ (corresponding to the polymer thickness), we obtain

2 $$z_{f}\left(d\right)=\frac{\ln\left[\mathrm{Kd}/\ln\left(1/\left(1-\phi_{c}\right)\right)\right]}{\mathrm{\mu ̅}}$$

where *K* characterizes the FPP kinetics. The critical (or lowest) irradiation dose *d*_c_, required for ϕ to first reach ϕ_c_ and thus *z*_f_ > 0, is obtained by rearranging eq 2, yielding $d_{c}=\frac{1}{K}\;\ln\frac{1}{1-\phi_{c}}$ . The interfacial width can be defined as *w* ≡ *e*/μ̅, characterizing the conversion gradient of the resulting asymmetric network.

### Effect of PEGDA Molecular Mass on FPP Kinetics

We investigated experimentally the influence of the molecular mass of the PEGDA precursor on FPP kinetics. Three PEGDA lengths are selected, with number-average molecular mass, namely, *M*_*n*_ = 250, 575, and 700 g mol^–1^, while maintaining a constant PI-to-PEGDA ratio (w/w) of 0.67% (corresponding to isopleth line ① in Figure 1d). Fourier transform infrared (FTIR) spectroscopy measurements (SI Section S1) were employed to determine the critical conversion ϕ_c_, which was found to be approximately the same across all three precursor PEGDA molecular masses (ϕ_c_ ≈ 0.2). To validate the applicability of the photoinvariant FPP model to this investigated system, the attenuation coefficient μ was measured under continuous UV irradiation. A constant value of μ was observed for irradiation doses below 700 mJ/cm^2^, which thus provides an upper bound for the photoinvariant regime (Figure 1e). Beyond this dose, photobleaching takes place and μ decreases with further exposure.

We characterize FPP front propagation kinetics by fabricating networks at varying irradiation doses *d* and measuring the resulting solid–liquid front position *z*_f_. Within the coarse-grained FPP framework,^21^ it follows from eq 2 that the inverse slope and the *z*_f_-intercept of a semilogarithmic plot of *z*_f_ vs *d* yield μ̅ and the critical irradiation dose *d*_c_, respectively. Experimental data show invariant slopes for all *M*_*n*_ in Figure 1f, indicating that μ̅ remains constant, with an average value of 3.4 ± 0.2 mm^–1^. This is expected, as the PI is the dominant absorbing species, and its concentration is constant across all three formulations, fixed at 0.0067 w/w, corresponding to 0.67% w/w PI/PEGDA. However, the *z*_f_-axis intercepts for three different *M*_*n*_ are clearly distinct, yielding different critical doses, namely, *d*_c_ = 21.3, 9.2, and 7.6 mJ/cm^2^, for *M*_*n*_ = 250, 575, 700 g mol^–1^, respectively. Higher *M*_*n*_ thus leads to a lower *d*_c_ (Figure 1f) or, equivalently, a lower induction time for solidification. Knowing ϕ_c_ and eq 2, we calculate the effective rate constant *K* for each oligomer based on Figure 1f: 0.011, 0.022, and 0.027 cm^2^ mJ^–1^ for *M*_*n*_ = 250, 575, and 700 g mol^–1^, respectively, following that higher *M*_*n*_ also leads to a higher front propagation rate, *K*. Interestingly, the velocity of FPP wave propagation is not well-defined as it varies over time (or *z*). We find that the traveling wave velocity is effectively constant for all *M*_*n*_ investigated, at constant *d*, and that the role of the precursor molecule *M*_*n*_ is simply that of changing *d*_c_. In simple terms, higher *M*_*n*_ benefit from an “earlier start” associated with their lower *d*_c_. At constant illumination dose (or time), networks originating from higher *M*_*n*_ thus grow taller, as the overall process is more “efficient.” We elaborate on these observations in SI Section S2, rationalized in terms of our minimal model.

### Nonmonotonic Dependence of FPP Kinetics on Photoinitiator Concentration

We next quantify the impact of the PI concentration on FPP kinetics for PEGDA oligomers of different *M*_*n*_. For this analysis, we select a series of PI:PEGDA (w/w), *R*_*w*_, to maintain constant PI:PEGDA stoichiometric ratios: *R*_*w*_ = 1.9, 1.56, and 0.68% for *M*_*n*_ = 250 g mol^–1^; 0.83, 0.68, and 0.29% for *M*_*n*_ = 575; and 0.68, 0.56, and 0.24% for *M*_*n*_ = 700 g mol^–1^ (see Figure 1d, isopleth line ②). These PI:PEGDA (w/w) ratios give molar ratios of 1:88, 1:107, and 1:248 (PI:PEGDA), respectively. Across all experiments (see Figure 2a–c and additional data available in SI Section S2), we find that increasing the PI:PEGDA (w/w) ratio results in decreased slope and *z*_f_-intercept, corresponding to a simultaneous increase in both μ̅ (mm^–1^) and *K* (cm^2^ mJ^–1^). Regardless of PEGDA *M*_*n*_, μ̅ scales linearly with *R*_*w*_, *viz*. μ̅ = 540.494 *R* + 0.12498 (Figure 2d). By contrast, while *K* also increases with the PI:PEGDA ratio (250 g mol^–1^: 1.11 *R*_*w*_, 575 g mol^–1^: 2.21 *R*_*w*_, and 700 g mol^–1^: 3.04 *R*_*w*_ [cm^2^ mJ^–1^]), the rate of increases depends on the specific precursor *M*_*n*_. The heat maps in Figure 2e–g summarize the interplay between the PI/PEGDA ratio and dose in terms of the resulting front position *z*_f_. Corroborating our findings above, greater *M*_*n*_ leads to higher *z*_f_ at comparable *d*, for all PI:PEGDA (w/w) ratios. A crossover in front propagation kinetics, from faster to slower, is observed upon increasing PI concentration. This nonmonotonic effect with PI arises from the shifting balance between two governing parameters μ̅ and *K*: low PI leads to a higher *d*_c_ (and lower *K*), meaning that frontal propagation starts at later times (or higher doses), but it also leads to a lower μ̅ (optical attenuation) and thus faster front propagation. As a result, under low PI conditions, small variations in exposure dose *d* lead to significant changes in *z*_f_, while under high PI conditions, *z*_f_ becomes less sensitive to *d*. This interplay highlights the importance of selecting appropriate PI concentrations and exposure *d* to optimize FPP processing (and associated economic and environmental trade-offs between PI loading and UV dose).

**Figure 2:**
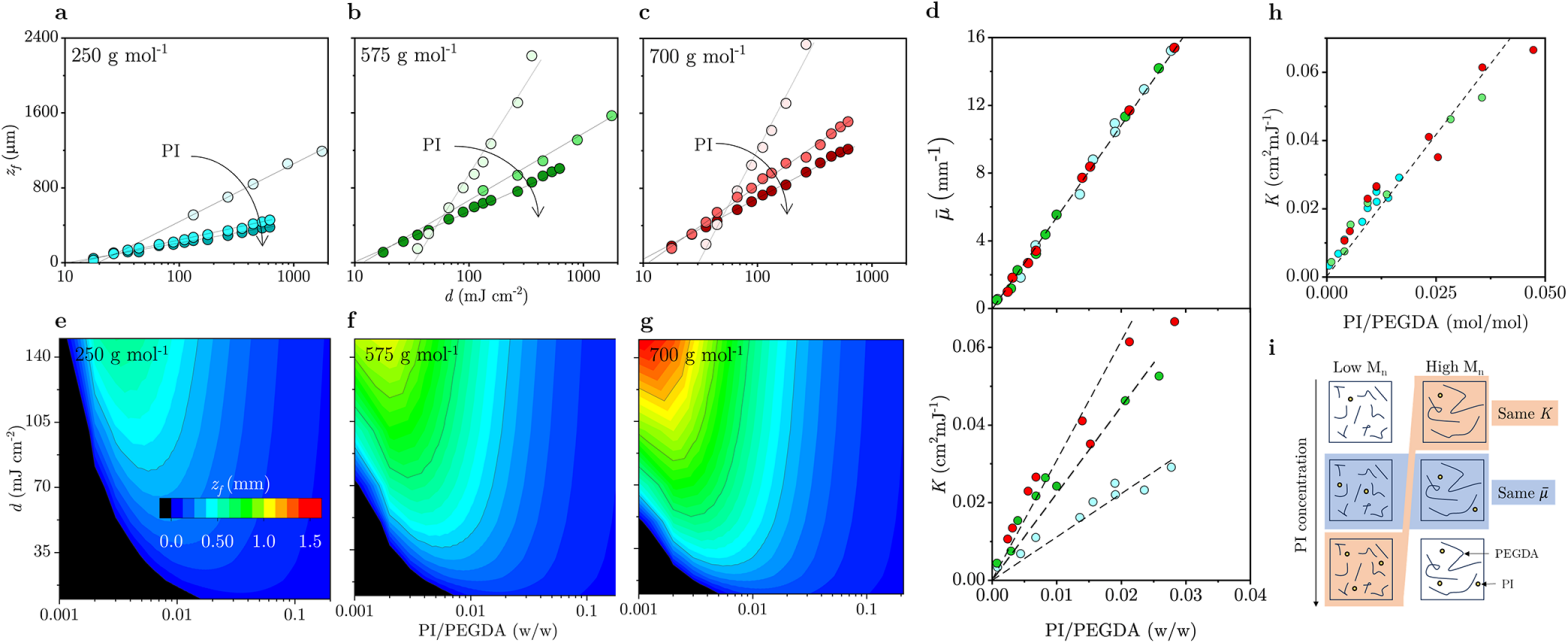
FPP kinetics measured in terms of front position *z*_f_ and irradiation dose *d* (mJ cm^–2^) and increasing photoinitiator concentration for (a) PEGDA *M*_*n*_ = 250 g mol^–1^, (b) 575 g mol^–1^ and (c) 700 g mol^–1^, corresponding to isopleth ② in Figure 1d. Lines are logarithmic fits to eq 2 yielding μ̅ and *K*. (d) Optical attenuation coefficient μ̅ increases linearly with PI/PEGDA (w/w) and collapses for all *M*_*n*_, while the FPP kinetics constant *K* increases with *M*_*n*_. (e–g) Heat map of *z*_f_ for different irradiation doses and PI:PEGDA ratios (w/w) for PEGDA *M*_*n*_ 250, 575, and 700 g mol^–1^. (h) Constant *K* effectively collapses for all *M*_*n*_ and constant PI:PEGDA stoichiometric ratio. (i) Illustration of the combined effects of PEGDA *M*_*n*_ and PI, including conditions for constant *K* at identical PI/PEGDA stoichiometric ratio, and constant μ̅ at fixed PI concentration.

### Unified *K* Values for Different *M*_*n*_ PEGDA

To understand why higher oligomer *M*_*n*_ results in faster polymerization kinetics, we consider instead the PI/PEGDA *molar ratio* (*R*_*m*_ ≡ PI (mol)/PEGDA (mol)) across different formulations and compute the corresponding *K* and μ̅ values. We observe that *K* scales linearly with *R*_*m*_ (*viz*. *K* = 1.66 *R*_*m*_ [cm^2^ mJ^–1^]) irrespective of precursor *M*_*n*_ (Figure 2h). This suggests that the FPP rate constant is governed primarily by the stoichiometric ratio of reactive species, namely, PI molecules and acrylate end groups, rather than by the chain length of the oligomer (Figure 2i). These results also corroborate the notion that the *K* parameter derived from the coarse-grained FPP model represents an effective rate constant, which encapsulates the overall conversion dynamics at the macroscopic level. It should not be interpreted as an intrinsic molecular rate constant of the reaction but rather as a system-level parameter reflecting the solidification kinetics of the FPP formulation.

### Modulating Pattern Curvature and Fluctuations with PEGDA Molecular Mass

We next consider the influence of PEGDA molecular mass on the self-assembly of FPP planar materials into 3D structures and their spatiotemporal response to environmental actuation. As outlined in Figure 3a, we fabricated and illustrated 2D starfish-shaped structures by UV patterning, subsequently removed from their (glass) substrates, and immersed them into a developing solvent (ethanol) for 3 min. At the stage of solvent immersion, we observe the emergence of curvature (κ ≡ 1/*r*, where *r* is the radius of curvature) and monotonic or fluctuating profiles, which we find to depend on *M*_*n*_. In this article, we define a minimal curvature fluctuation as the sequential transition κ > 0 → κ ≤ 0 → κ > 0. The polymer network is then removed from ethanol and exposed to ambient conditions, where κ dynamics re-emerge.

**Figure 3:**
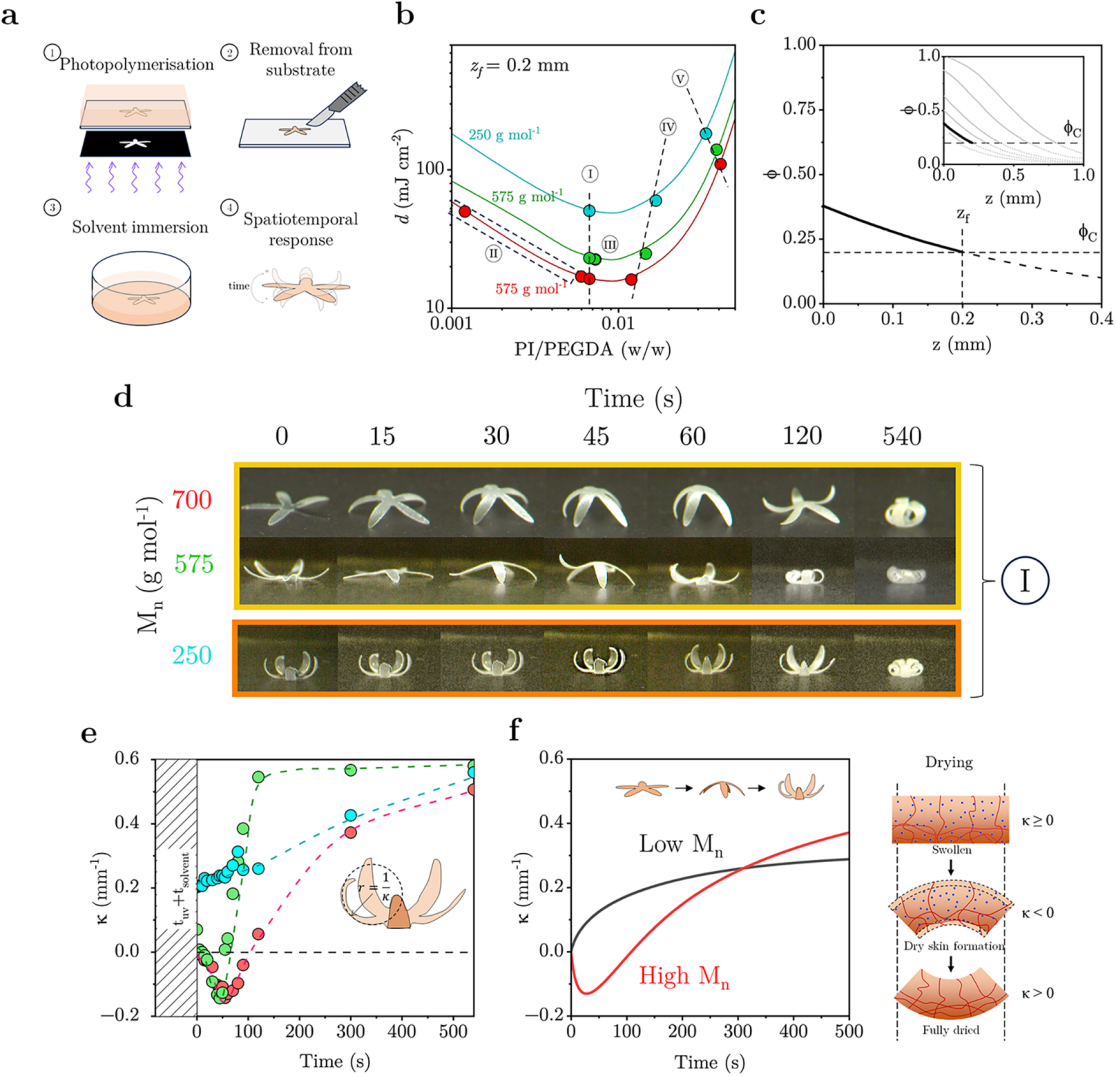
(a) Schematic of FPP fabrication and solvent development, depicting photopolymerization, substrate removal, solvent immersion, and spatiotemporal response. (b) Experimental map of PI/PEGDA (w/w) and irradiation dose *d* (mJ cm^–2^) investigated, defining isopleths Ⓘ to ⓥ, yielding a *constant* pattern thickness *z*_f_ = 0.2 mm. (c) FPP conversion ϕ­(*z*) for isopleth Ⓘ, corresponding to constant PI/PEGDA (w/w) = 0.0067, and μ = 3.6 mm^–1^ at *z*_f_ = 0.2 mm. The inset illustrates ϕ­(*z*, *t*) for higher irradiation doses, until full conversion of the skin layer. (d) Time-dependent 4D FPP structures obtained for a “starfish” pattern at isopleth Ⓘ, showing a nonlinear evolution of curvature κ. (e) Evolution of κ computed from images in (d). (f) Computed time-dependent κ profile and illustration of the fluctuation mechanism and associated drying of asymmetric FPP networks. Data for *M*_*n*_ 700 g mol^–1^ have been previously reported^33^ and are included for comparison.

We first examined a constant PI:PEGDA weight ratio of 0.67% (Figure 3b, isopleth line Ⓘ) to ensure the same μ̅. We fabricate starfish-shaped structures with different oligomers *M*_*n*_ and select *d* to achieve a constant *z*_f_ ≈ 0.2 mm. These operating conditions ensure consistent ϕ­(*z*) profiles, as depicted in Figure 3c.

Given that FPP innately yields asymmetric networks along the *z*-direction (normal to film surface), the spontaneous emergence of curvature can be expected due to a stress differential across the two facets, at *z* = 0 and *z* = *z*_f_, due to the cross-linking gradient. In ambient air, the asymmetric networks fabricated from PEGDA with different *M*_*n*_ values exhibit a distinct spatiotemporal response (Figure 3d,e). Networks formed with *M*_*n*_ = 250 g mol^–1^ show a monotonic increase of curvature over time (κ > 0, bending toward low ϕ). However, networks fabricated from *M*_*n*_ = 575 and 700 g mol^–1^ display κ fluctuations in curvature during the drying process, from κ ≥ 0 (nearly flat), to κ < 0 (bending toward high ϕ facet), and then back to κ > 0. These observations show that oligomer *M*_*n*_ plays a significant role in governing the structural dynamics and responsiveness of the printed material.

The origin of curvature fluctuations in photopolymerized PEGDA structures has previously discussed.^33^ In short, these are caused by evaporation–diffusion of the solvent, which impacts the mechanical stress distribution evolution over time. Upon immediate exposure to ambient conditions, the swollen polymer begins to dry, rapidly forming a thin, stiff outer layer due to surface evaporation. This dry skin induces interfacial stresses against the softer, solvent-rich interior, leading to an initial negative curvature. The effect is most pronounced when the base of the structure (more cross-linked) experiences greater compressive stress than the less cross-linked front interface. As drying progresses, the solvent diffuses outward from the interior, gradually increasing stiffness throughout the polymer. This redistribution of internal stress eventually reverses the curvature, resulting in a final positive bending as the system approaches equilibrium.

To explore this phenomenon, we employ a minimal modeling framework^33^ based on the coupling of evaporation–diffusion phenomena according to Fick’s second law and its boundary conditions at *z* = 0 (illuminated surface) and *z* = *z*_f_ (solid–liquid interface) to compute the spatiotemporal profile of the solvent concentration along the polymer network

3 $$\frac{\partial C\left(z,t\right)}{\partial t}=\frac{\partial }{\partial z}\left(D_{e}\frac{\partial C\left(z,t\right)}{\partial z}\right)$$

where *D*_e_ is an “effective” diffusion constant, and *t* is the time following exposure to ambient air, imposing Robin boundary conditions

4 $${-D_{e}\frac{\partial C\left(z,t\right)}{\partial z}|}_{z=0}=k_{\mathrm{evap}}\left[C\left(0,t\right)-C_{\mathrm{air}}\right]$$

5 $${-D_{e}\frac{\partial C\left(z,t\right)}{\partial z}|}_{z=z_{f}}=-k_{evap}\left[C\left(z_{f},t\right)-C_{air}\right]$$

for the rate of solvent loss at both interfaces, *z* = 0 and *z* = *z*_f_ (analogous to common heat loss conditions due to convection at a surface).^38^ We compare two representative cases of low and high PEGDA molecular masses, with simulation parameters summarized in SI Table S1. The high-*M*_*n*_ polymer network is capable of absorbing more solvent and undergoing greater swelling (see Figures S5 and S6), which we associate to its larger network mesh size. While ϕ­(*z*) profiles are imposed to be constant in all *M*_*n*_ cases (ensured by identical μ̅ and ϕ_c_ values), the resulting mechanical response differs considerably. Building upon the model of Wang et al.,^39^ we describe the evolution of the Young’s modulus *E* during evaporation as

6 $$E\left(z,t\right)=E_{0}+\left[E_{c,\mathrm{swollen}}\left(\phi_{c},\eta_{\mathrm{evap}}=0\right)+\left(E_{c,\mathrm{dry}}-E_{c,\mathrm{swollen}}\right){\left(1-\eta_{\mathrm{evap}}\right)}^{n_{1}}\right]{\left[\phi -\phi_{c}\right]}^{n_{2}}$$

where *E*_0_ is the modulus of the neat network formed during photopolymerization upon reaching ϕ_c_, *E*_c_ corresponds to the critical Young’s modulus at fully dry (η_evap_ = 0) or swollen condition (η_evap_ = 1), and *n*_1_ and *n*_2_ correspond to the power law exponents for η_evap_ = *C*(*z*, *t*)/*C*(*z*, *t* = 0) and ϕ (within the range of 1.8–2.3, used as fitting parameters). Further, the curvature κ is quantified by computing the ratio of bending moment and bending stiffness as shown below

7 $$\kappa \left(t\right)=\frac{\int_{0}^{z_{f}}E\left(z,t\right)\varepsilon \left(z-z_{N}\left(t\right),t\right)dz}{\int_{0}^{z_{f}}E\left(t\right){\left(z-z_{N}\left(t\right)\right)}^{2}dz}$$

where ε denotes strain and the position of the neutral axis *z*_N_ can be quantified as

8 $$z_{N}\left(t\right)=\frac{\int_{0}^{h}E\left(z,t\right)zdz}{\int_{0}^{h}E\left(z,t\right)dz}$$

This minimal model can capture the overall behavior (Figure 3f) observed in experiments and provides mechanistic insights into the *M*_*n*_ dependence of the curvature response. Details of simulation results are provided in Figure S9.

We rationalize the observations in terms of the higher cross-link density of lower precursor *M*_*n*_ networks, resulting in greater initial Young’s modulus *E*_0_ at the critical threshold (ϕ = ϕ_c_) (see Figure S7). In turn, this increased stiffness reduces the mechanical contrast across the material interfaces, thus, reducing the internal stress gradient σ across the thickness. The generation of negative curvature can thus be expected to be reduced or suppressed in lower *M*_*n*_ structures, which aligns with our experimental observations of the absence of curvature fluctuation, contrasting with higher *M*_*n*_ counterparts (Figure 3f).

### Unified Fabrication Framework for Nonplanar FPP Networks

We finally investigate the influence of varying μ̅ across three different oligomer precursor *M*_*n*_ at constant *z*_f_ = 0.2 mm, as shown in Figure 4a, with the PI:PEGDA (w/w) ratio based on Figure 3b, isopleth line 
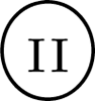
–ⓥ, on spatiotemporal response of networks (additional data is available in Figure S8). In this series, we vary μ̅ via different PI:PEGDA ratios (w/w) and, to keep *z*_f_ constant, we adjust exposure dose *d*. Figure 4b shows the corresponding ϕ­(*z*) profiles for all experiments shown in Figures 3d, 4a, and S5 (note that ϕ profiles for three different oligomer *M*_*n*_ with 0.67% PI:PEGDA w/w ratio overlap, hence the orange dashed line is provided to show different 3D/3D fluctuation outcome). In the network with higher oligomer *M*_*n*_, when Δϕ ≡ ϕ­(*z* = 0) – ϕ­(*z*_f_ = 0.2 mm) (≈0.14–0.2), we observe 3D fluctuations. Outside of this range, regardless of *M*_*n*_, the resulting structures are either flat (2D) when Δϕ ≈ 0.8, or form 3D shapes without curvature fluctuation Δϕ ≈ 0.36–0.55 or Δϕ ≈ 0.02. However, for networks comprising low oligomer *M*_*n*_, we found that across all Δϕ, the 3D flucuations are absent. Evidently, both μ̅ and network *M*_*n*_ dictate the dynamic response of FPP asymmetric networks, in terms of 2D, 3D, and 3D fluctuations at constant material thickness in the map depicted in Figure 4c (illustrated here for *z*_f_ = 0.2 mm). We observe that 3D structures exhibit curvature fluctuations emergence in high oligomer *M*_*n*_ (575 and 700 g mol^–1^) within a limited range of μ̅ ≈ 1.8–5.0 mm^–1^. In other ranges of μ̅ ≈ 5.0–12.0 mm^–1^ and μ̅ ≤ 1.5 mm^–1^, the networks establish a 3D structure but do not show κ fluctuations. However, at μ̅ ≥ 12.0 mm^–1^, the networks do not assemble into a 3D structure (2D, κ ≤ 0.02 mm^–1^). By contrast, networks with low oligomer *M*_*n*_ (250 g mol^–1^) do not show any κ fluctuations within the experimental range of μ̅.

**Figure 4:**
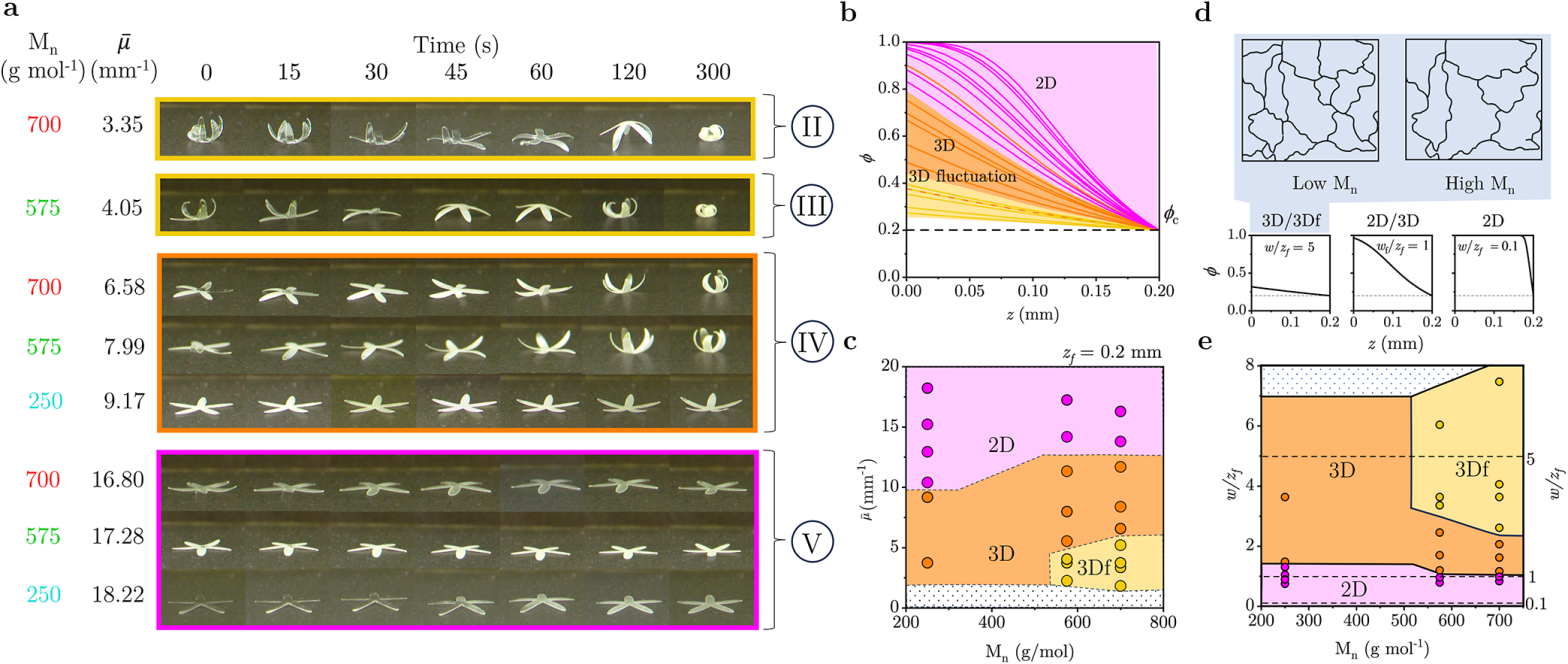
(a) 4D FPP origami “starfish” structures obtained with different oligomer *M*_*n*_ and μ̅, corresponding to isopleths Ⓘ-ⓥ in Figure 3b (detailed in the SI). (b) Overlaid conversion profiles ϕ­(*z*) corresponding to all isopleths Ⓘ-ⓥ and SI Data S5, at constant *z*_f_ = 0.2 mm, color-coded according to the morphotemporal response: planar (purple), 3D structure (orange), 3D curvature fluctuation (yellow). The orange dashed line inside the yellow area indicates a system of *M*_*n*_ 250 g mol^–1^ that does not fluctuate. (c) Overall morphological response map of FPP origami in terms of *M*_*n*_ and μ̅. (d) Illustrative conversion profile ϕ­(*z*) with various interfacial widths to pattern thickness ratios, *w*/*z*_f_. The network schematics depict the cross-linking density of low- and high-molecular-weight oligomers (*M*_*n*_), which dictates the 3D/3D curvature fluctuation behavior. (e) Morphological response map of FPP origami in terms of interfacial width *w* normalized with *z*_f_ and *M*_*n*_.

By adjusting μ̅, the mechanical properties across the network are altered according to the ϕ profile (Figure 4b). At high μ̅, where ϕ ≳ 0.8 at *z* = 0 across all oligomer *M*_*n*_, the Young’s modulus *E* scales as a power of ϕ. This eventually increases the resistance to bend near *z* = 0, thereby suppressing curvature formation κ. For the emergence of 3D structures, the overall bending moment must be significant relative to resistance to bending.

Within the FPP framework, varying optical attenuation μ̅ governs the ϕ profile, and in turn, the interfacial width *w*, that is a metric for the steepness of the conversion gradient, as illustrated in Figure 4d. It is therefore tempting to compare the two length scales, *w* and *z*_f_, and we thus obtain the morphological map in terms of a dimensionless interfacial width, *w*/*z*_f_, shown in Figure 4e. This representation is analogous to that of Figure 4c but provides further clarity in that, for instance, 2D structures are found for *w*/*z*_f_ ≲ 1, effectively when the interfacial width is commensurate or smaller than the pattern thickness (and therefore step-like conversion profiles). By contrast, 3D structures emerge for interfacial widths much broader than the pattern thickness, and thus smooth conversion profiles.

## Conclusion

Our study demonstrates that oligomer molecular mass is a critical and easily tunable parameter in the design of 4D materials fabricated via frontal photopolymerization (FPP). By varying the chain length of a model difunctional acrylate oligomer, PEGDA, we isolated the influence of precursor *M*_*n*_ from FPP process conditions (optical attenuation and light exposure) to elucidate its impact on both polymerization kinetics and shape transformations.

Our findings show that while absolute polymerization kinetics vary with *M*_*n*_, we find that the velocity of the propagating fronts are effectively unchanged but that higher *M*_*n*_ yields a lower critical threshold exposure and thus an earlier onset which results in faster kinetics at comparable time scales (or doses). These kinetics are governed primarily by the oligomer-to-photoinitiator ratio, indicating stoichiometric control over the front propagation behavior. However, the oligomer-to-photoinitiator ratio also dictates the competing relationship between *K* and μ̅, which results in a nonmonotonic, albeit predictable, overall reaction rate. This insight provides a framework for the rational formulation design of FPP systems.

Moreover, we find that the solvent development of FPP networks can induce complex morphological responses, including curvature and fluctuating curvature, that also depend on *M*_*n*_. Our minimal model of evaporation–diffusion coupled to the FPP equations can effectively capture the experimental observations and provide a useful design framework for FPP curvature dynamics. In general, higher *M*_*n*_ precursors yield networks with larger mesh dimensions (at constant thickness *z*_f_) which support complex, nonmonotonic curvature evolution induced by solvent development. In contrast, lower molecular mass systems produce tighter networks that deform monotonically, as expected from the conversion (and stress) gradients across the thickness. Interfacial widths *w* larger than pattern thickness *z*_f_ are needed for the emergence of 3D behavior. Overall, our findings demonstrate how *M*_*n*_ can dictate the emergence, directionality and magnitude of pattern curvature κ, and its possible evolution in time. Our approach provides a facile design strategy for programming shape change into gradient polymer network materials without altering fabrication processes or external stimuli. When combined with the inherent gradient-forming capabilities of FPP, this approach offers a scalable, single-step route to structurally encoded 4D systems with potential applications in soft robotics, deployable biomedical scaffolds, adaptive optics, and beyond.

## Supplementary Material



## Notes

FTIR for ϕ_c_ measurement; FPP model of traveling waves and additional kinetics data; solvent mass uptake for different PEGDA oligomer *M*_*n*_; details of the simulation model, parameters, and results; and additional data on spatiotemporal responses on printed polymers (PDF)
