# Supplementary material for: Curvature Dynamics of PEGDA Asymmetric Networks via Frontal Photopolymerization: Effect of Chain Length and Optical Attenuation
Source: Macromolecules. 2026 Feb 12;59(4):1978–86. doi: 10.1021/acs.macromol.5c02783 (PMC12947670; doi:10.1021/acs.macromol.5c02783)
Supplement: Supplementary file 1 [file ma5c02783_si_001.pdf]

Supplementary Information:

Curvature dynamics of PEGDA asymmetric networks via frontal photopolymerization: effect of chain length and optical attenuation

Muhammad Ghifari Ridwan,[a] Huseyin Mirac Dizman,[a,b] Isobel Bentley,[a] Alessandra Vitale,[b] and João T. Cabral*[a]

[a]*Department of Chemical Engineering, Imperial College London, London SW7 2AZ, United Kingdom*

[b]*Department of Applied Science and Technology, Politecnico di Torino, Torino, Italy*

***Email:** j.cabral@imperial.ac.uk

S1 Differential crosslinking by FTIR

FPP model comprises many physical parameters, readily measured via different experiments. One of the physical parameters required is critical monomer to polymer conversion ϕ_c. We measure ϕ_C via Fourier-Transform Infrared (FTIR) spectroscopy measurement. We specifically investigate the conversion C=C bond in acrylate groups around wavenumber of 1600-1650 cm^{-1} for three different samples: monomer, polymer front, and polymer base. To ensure consistent measurements across different parts and samples, we normalize C=C peak with a reference peak, C=O bond around wavenumber of 1700-1750 cm^{-1}. Intuitively, as the photopolymerization progresses, the quantity of C=C bond is reduced, to form a crosslinking bond, connecting one monomer with others. However, the quantity of C=O bonds remains, due to exclusion from overall photopolymerization reaction. We fabricate the sample with PI:PEGDA w/w ratio of 0.67%, and $z_f = 0.2$ mm. The fabricated sample is pad-dried to remove excess monomer on the surface. We then measure the infrared (IR) absorbance of the sample on the front, base and its monomer (Fig. S1).

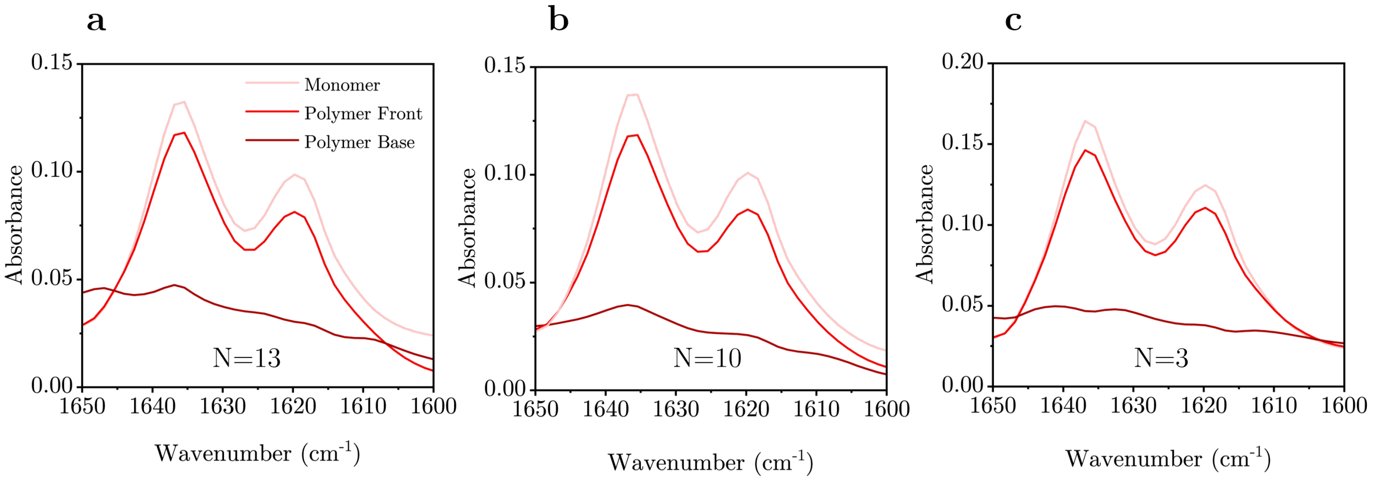

Figure S1 Fourier Transform Infrared (FTIR) absorbance spectra from 1600 to 1650 cm^{-1}, correspond to C=C bond in acrylate groups in PEGDA, for three different samples: liquid monomer, polymer front, and polymer base. The samples were fabricated using three different monomer number-average molecular weight M_n, which corresponds to different number of ethylene oxide repeating monomer (N). The samples are consistently fabricated with 0.67% PI:PEGDA ratio at different irradiation dose. (a) M_n 700, N 13, $d = 227$ mJ cm^{-2}, (b) M_n 575, N 10, $d = 275$ mJ cm^{-2}, and (c) M_n 250, N 3, $d = 660$ mJ cm^{-2}.

To calculate the ϕ_c for networks with different monomer M_n, we first evaluate the absolute critical monomer-to-polymer conversion, $\chi_c = 1 - \frac{A^{front}_{C=C}/A^{front}_{C=O}}{A^{monomer}_{C=C}/A^{monomer}_{C=O}}$, where $A^{front}_{C=C}$, $A^{front}_{C=O}$, $A^{monomer}_{C=C}$, and $A^{monomer}_{C=O}$, correspond to the area under the absorbance curve of C=C and C=O bonds for front and base of the network.[1,2] We then compute the absolute maximum monomer-to-polymer conversion, $\chi_{max} = 1 - \frac{A^{base}_{C=C}/A^{base}_{C=O}}{A^{monomer}_{C=C}/A^{monomer}_{C=O}}$, from networks fabricated with $d = 100$mJ cm^{-2}. Finally, we determine the relative critical monomer-to-polymer conversion, $\phi_c = \frac{\chi_c}{\chi_{max}}$The results show that networks with different M_n approximately have the same ϕ_c values, 0.2.

S2 Frontal Photopolymerization (FPP) Traveling Waves

In this section, we provide details about coarse-grained model of frontal photopolymerization (FPP). Frontal photopolymerization is a photoinduced polymerization sustained via continuous light exposure, triggering a directional solidification process, separating crosslinked network with unreacted monomer called a front.[3,4] In the limiting case of photoinvariant process ($\bar{\mu} \approx \mu_0$) process, the monomer-to-polymer conversion ϕ profile conforms a traveling wave profile dictated by $\bar{\mu}$. The traveling wave profiles can be expressed by:[3]

$$\phi(z,d) = 1 - \exp[-Kd\exp\{(-\bar{\mu}z)\}] \tag{S1}$$

where K, and d correspond to monomer-to-polymer conversion rate and irradiation dose. While the shape of ϕ profile is prescribed by the $\bar{\mu}$, the spatial position of ϕ profile is determined by d. To illustrate this, we can see that the shape of ϕ profiles at various d is shape-invariant. Different values of d spatially define the stopping point of the traveling waves (Fig. S2). By comparing the monomer M_n and its polymerization kinetics, the traveling wave stopping point is differ. The higher K value corresponds to further stopping point, or network thickness (z_f).

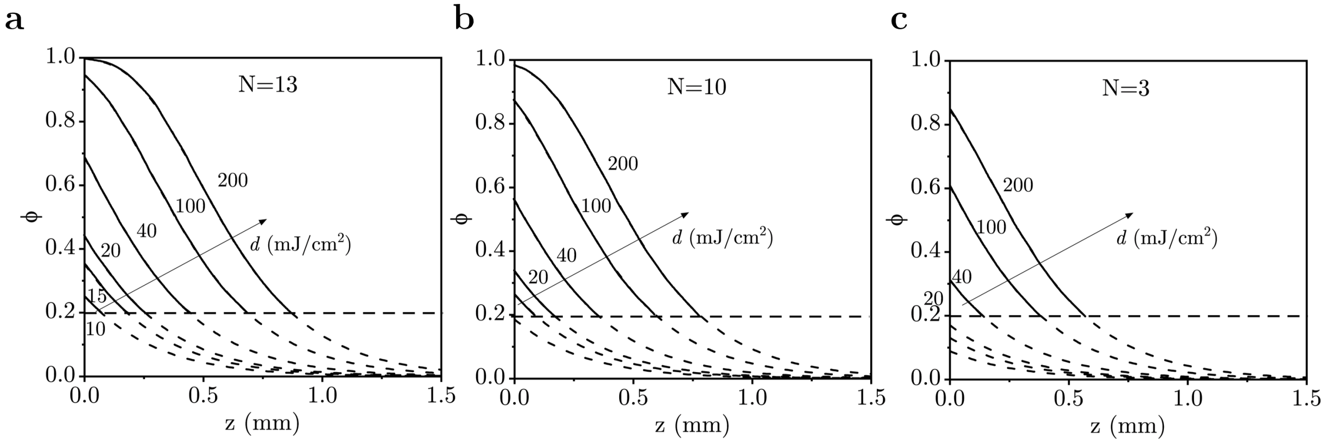

Figure S2 Conversion profile ϕ of PEGDA network with different irradiation dose d (10, 15, 20, 40, 100, and 200 mJ/cm^2) for three different number-average molecular weights (M_n), which correlate with ethylene oxide repeating monomer (N): (a) M_n 700: 13, where all selected doses d forms a solidified networks. (b)M_n 575: where doses of 15, 20, 40, 100, and 200 mJ/cm^2 form solidified networks. (c) M_n 250: where doses of 40, 100, and 200 mJ/cm^2 form solidified networks. All ϕ profiles across different d and monomer M_n are shape-invariant.

We then compared the velocity of the traveling waves by taking the first derivative of $z_f(d)$ ($\frac{dz_f}{dd}$) equation against d and z. We compared two systems, a series of constant $\bar{\mu}$ and varying K, and vice versa. In the first system, we maintain $\bar{\mu}$ at 3.4 mm^{-1} with K from 0.01 to 0.03 mJ cm^{-2}, while in the second system, K is kept constant at 0.01 mJ cm^{-2} and $\bar{\mu}$ are 1, 5, and 10 mm^{-1}.

In the first system (constant $\bar{\mu}$ and varying K), we observe exactly same profiles for $\frac{dz_f}{dd}$ against d, except for

the initial point (Fig. S3). The initial points on d-axis indicate d_c, which decreases with increasing K. Note that we have separately demonstrated the linear proportionality of K with M_w, within the range investigated (Fig. 1f of the main paper). We infer that the same profile corresponds to the same velocity of FPP traveling waves. However, a higher K (or M_w) leads to an 'earlier start'. While the front velocity is unchanged at fixed dose (or time), it clearly varies at fixed patterned height, as illustrated in Fig S3b.

In the second system (constant K and varying $\bar{\mu}$), we observe different profiles for $\frac{dz_f}{dd}$ against d, except initial points. The same initial points on d-axis indicate same d_c. However, the traveling waves do not propagate at the same velocity. In addition, for both systems, $\frac{dz_f}{dd}$ relative to z exhibits different velocities at any z point.

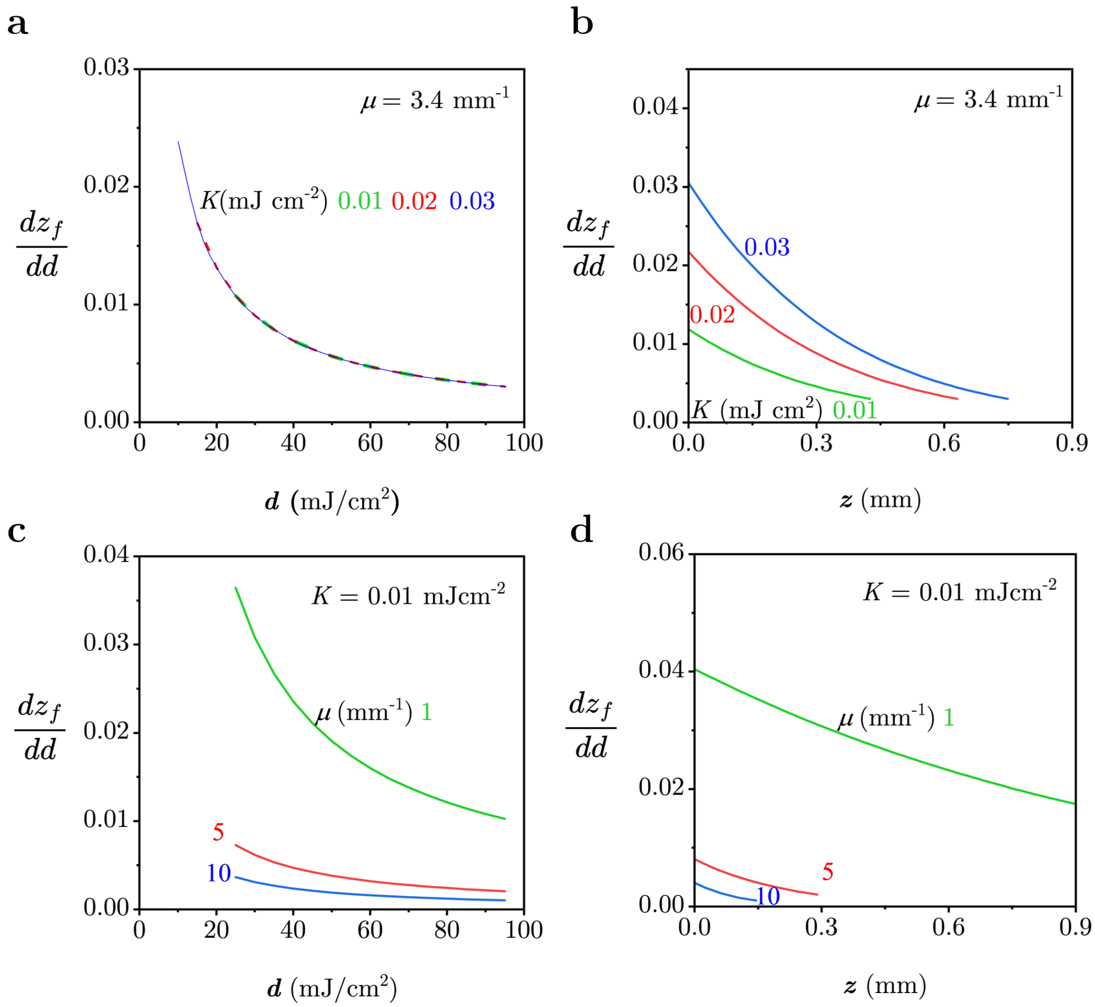

Figure S3 Detailed kinetic analysis for three different number-average molecular weights (M_n), which correlate with ethylene oxide repeating monomer (N): First derivative of z_f against d with varying K and constant $\bar{\mu}$, reflecting the front velocity plotted against (a) d, (b) z and first derivative of z_f against d with constant K and varying $\bar{\mu}$, reflecting the front velocity plotted against (c) d, (d) z.

Further, we provide additional data on kinetic characterization of PEGDA with different monomer M_n across different PI:PEGDA (w/w) ratios. We exposed the mixture of PEGDA monomer and PI with UV light (365 nm)

with different exposure times, resulting in different network thickness z_f. Before the data acquisition, the excess unreacted resin is removed via pad drying. In figure S4, $\bar{\mu}$ (inverse slope of the graph) and K ($K = \frac{1}{y_{x=0}} \ln \frac{1}{1-\phi_c}$), across all monomer M_n, are increasing with the higher PI content, indicating faster conversion rate but limited light penetration.

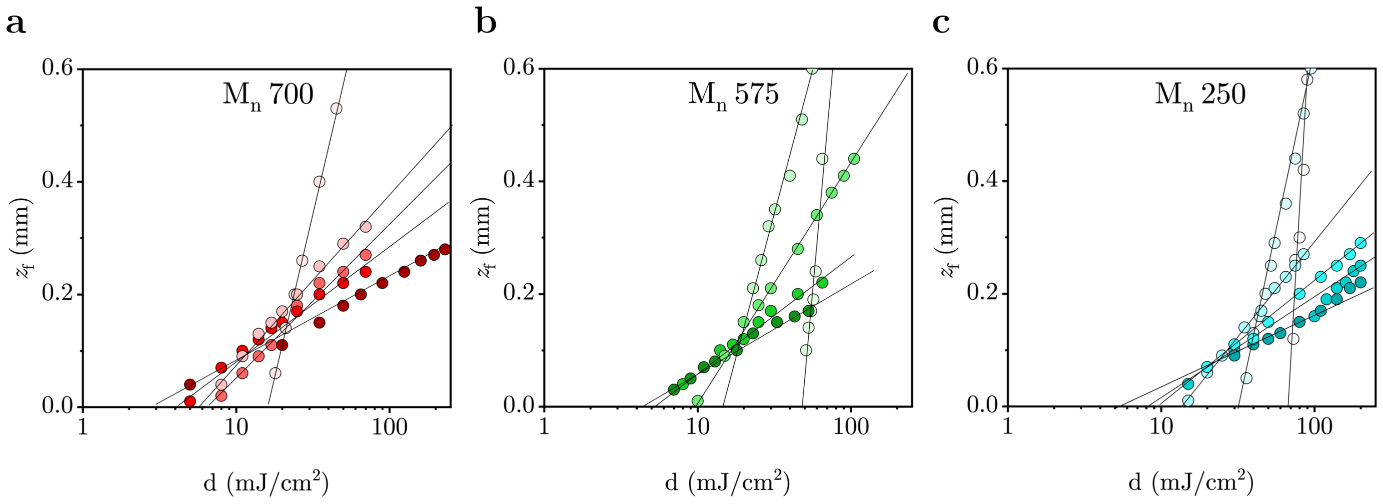

Figure S4 Kinetic characterisations for three different monomer number-average molecular weights (M_n [g/mol]) with different PI:PEGDA (w/w) ratios. (a) M_n 700: 0.32%, 1.4%, 1.5%, 2.1%, 2.8%, (b)M_n 575: 0.07%, 0.54%, 1.0%, 2.1%, 2.6%, (c) M_n 250: 0.08%, 0.44%, 1.3%, 1.9%, 2.3%, 2.8%.

Here, we tabulated all kinetic characterization data from the main text and supplementary information:

Table S1 Tabulated value for K and $\bar{\mu}$

M_n (g mol^{-1})	PI/PEGDA w/w (%)	$\bar{\mu}$ (mm^{-1})	K (cm^2 mJ^{-1})
0.0838	250	0.55148	0.0033
0.441	250	1.83466	0.00683
0.675	250	3.72426	0.011
1.359	250	6.74445	0.01618
1.564	250	8.79121	0.02018
1.91	250	10.4296	0.02205
2.357	250	12.93661	0.02322
2.776	250	15.2207	0.02912
0.076	575	0.50888	0.00442
0.293	575	1.17938	0.00753
0.396	575	2.25327	0.01536
0.68	575	3.22581	0.02172
1.00	575	5.54	0.02423
2.063	575	11.33787	0.04625
2.586	575	14.1844	0.05258
0.241	700	0.9901	0.01065
0.316	700	1.81587	0.01345
0.559	700	2.68817	0.02296
0.679	700	3.40669	0.0266
1.398	700	7.7101	0.04105
2.129	700	11.69591	0.06142
2.827	700	15.38462	0.06659

S3 Solvent absorption in polymer network with different crosslinking density

To estimate the distribution of the solvent concentration across asymmetric networks with different monomer M_n, we conduct solvent absorption measurement with networks with PI:PEGDA w/w composition of 0.67%. We fabricate the networks with varying d, resulting in four different z_f for each monomer M_n. Upon UV irradiation exposure, the network is pad-dried, and thus the mass is measured ($m_{monomer+networks}$, which equals to mass of networks and excess monomer). Then the network is immersed in solvent for 3 min. The sample mass after solvent immersion is quantified (assumed to be $m_{solvent\ immersion}$ mass of networks and the mass of solvent). Further, the sample is left in ambient air for 2 h, and the weight of the sample is evaluated (m_{dried}, mass of networks). The solvent absorption in polymer network is calculated via $m_{solvent} = \frac{m_{solvent\ imersion} - m_{dried}}{m_{dried}}$. The results of solvent absorption experiments are shown in Fig. S5 below:

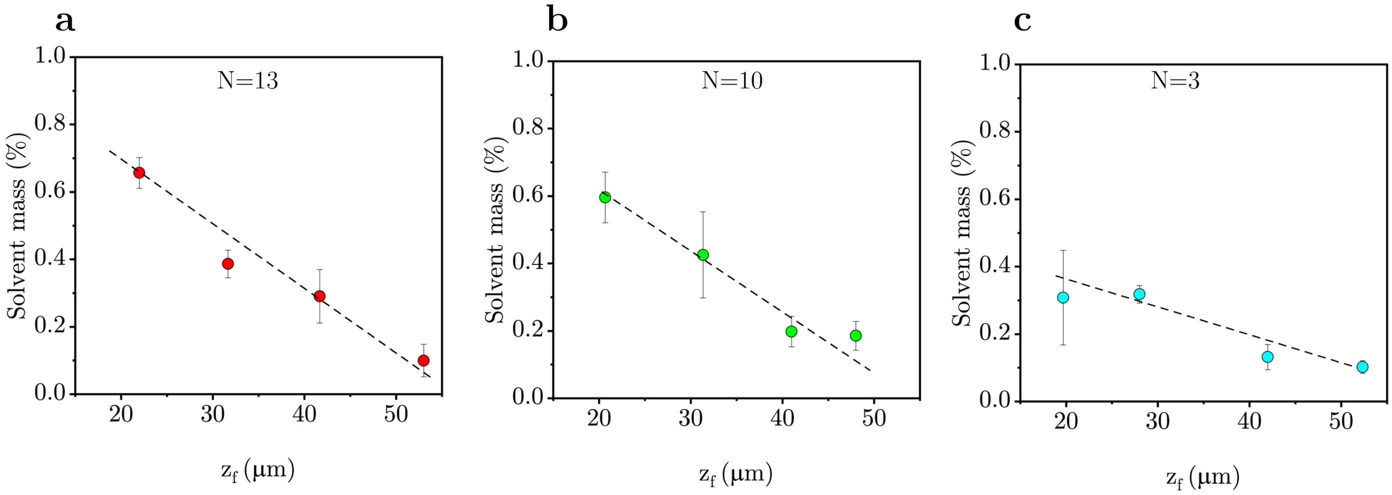

Figure S5 Solvent (ethanol) absorption capacity in PEGDA across different sample thicknesses for three different monomer M_n, which correspond to numbers of ethylene oxide repeating monomer (N): (a) M_n 700, N 13 ($m_{solvent} = -1.91039 \times z_f + 1.08$), (b) M_n 575, N 10 ($m_{solvent} = -1.81 \times z_f + 0.981$), and (c) M_n 250, N 3 ($m_{solvent} = -8.31 \times z_f + 0.530$).

The results indicate decreasing trends in solvent mass absorption experiments across three different monomer M_n. Intuitively, the higher the ϕ, the less the solvent absorption capacity, due to limited free volume and tighter architecture, limiting the capability to swell. In terms of networks with different monomer M_n, the network mesh size is dictated by the monomer size. The shorter the monomer length, the smaller the mesh size. Consequently, the networks have smaller free volume, hence, limiting capability to absorb solvent.

S4 Details of evaporation-diffusion simulation on gradient polymer network

We follow simple physico-mechanical framework to unravel the difference between networks of low and high oligomer M_n.[5] The simulation accounts for differential crosslinking across the polymerized network, calculated via FPP coarse-grained model.[3] The crosslinking gradient of polymerized networks gives rise to variability of the polymer network mechanical properties across its thickness. To simulate the curvature during the drying process, we account simultaneously two phenomena: diffusion and evaporation processes. These two processes further affect the local mechanical properties of the polymer network. The foundation of the curvature simulation lies on three theories, (1) FPP coarse-grained model, (2) Fick's second law, and (3) Bernoulli's beam theory. To capture the spatiotemporal evolution of ϕ, we use equation S1. We then solved Fick's second law :

$$\frac{\partial C(z,t)}{\partial t} = \frac{\partial}{\partial z}\left(D_e \frac{\partial C(z,t)}{\partial z}\right) \tag{S1}$$

where C, t, and D_e are concentration, time, and effective diffusion constant. The diffusion constant in gradient polymer is not constant. Then, we introduce the variability in diffusion constant by $D_e = D_0\phi^{-\alpha}(z)$.[6] To solve equation S1, we define initial condition by

$$C(z,0) = C_0\phi^{-n}(z) \tag{S2}$$

and boundary conditions:

$$-D_e \frac{\partial C(z,t)}{\partial z}\bigg|_{z=0} = k_{evap}[C(0,t) - C_{air}] \tag{S3}$$

$$-D_e \frac{\partial C(z,t)}{\partial z}\bigg|_{z=z_f} = -k_{evap}[C(z_f,t) - C_{air}] \tag{S4}$$

By numerically solving (finite volume method, where Forward Euler for time and central difference discretizations are employed) eq. S1 with its respective initial (eq. S2) and boundary conditions (eq. S3,S4), we obtain the concentration profile $C(z,t)$. By mass balance, $C(z,t) + C_{evap}(z,t) = C(z,0)$, we define the evaporation ratio η_{evap} as:

$$\eta_{evap} = \frac{C(z,t)}{C(z,0)} \tag{S5}$$

Next, we define the mechanical response of the polymer network towards drying from the fully swollen state. To begin with, we need to understand the strain ε relations with the ϕ profile in the network. We follow the relations between swelling and desolvated strain in the previous work for the fully swollen and dried state.[7,8] The equations are shown below:

$$\varepsilon_{swell} = \left[\frac{g}{\phi^{0.6} - 1 + g}\right]^{1/3} - 1 \tag{S6}$$

$$\varepsilon_{dry} = [1 - h(1 - \phi)] - 1 \tag{S7}$$

where ε_{swell}, ε_{dry}, g, and h, are strain at fully swollen state, strain at fully dried state, solvent-polymer swelling coefficient, and shrinking constant, respectively. Next, we consider partial swelling/dried networks and correlate with the resultant ε and Young's modulus E. The resultant equation for ε is shown below:

$$\varepsilon = (\varepsilon_{swell} - \varepsilon_{dry})(1 - \eta_{evap}) + \varepsilon_{dry} \tag{S8}$$

We then plot the resultant ε for two networks from different monomer M_n by varying η_{evap} (Fig. S6). The plot shows that higher M_n has larger spectrum of ε compared to network comprises of lower monomer M_n.

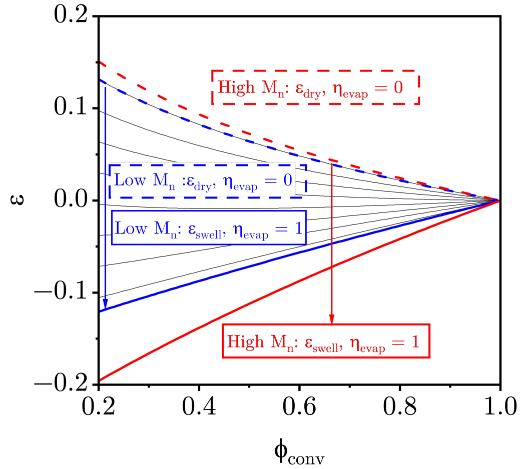

Figure S6 Strain value for PEGDA used in simulations. The solid red, blue and black lines correspond to strain of low molecular weight PEGDA maximum, minimum, and variable evaporation ratio η_{evap}. The dash red and blue lines correspond to maximum and minimum strain of high molecular weight.

Further we set out the resultant E across different ϕ and η below[9]:

$$E = E_0 + [E_{c,swollen} + (E_{c,dry} - E_{c,swollen})(\eta_{evap})][\phi - \phi_c] \tag{S9}$$

We observe a contrasting behaviour of the E spectrum with ϕ, to that of η compared with ε. In this case, networks from lower monomer M_n show a larger span of E value compared to higher monomer M_n. Intuitively, this is due to the difference in crosslinking density. The smaller chain length of monomer M_n consequently will have more crosslinking site in the same unit volume compared to a longer chain length monomer. The higher crosslink density resulting in mechanically stronger network, including in E (Fig. S7).

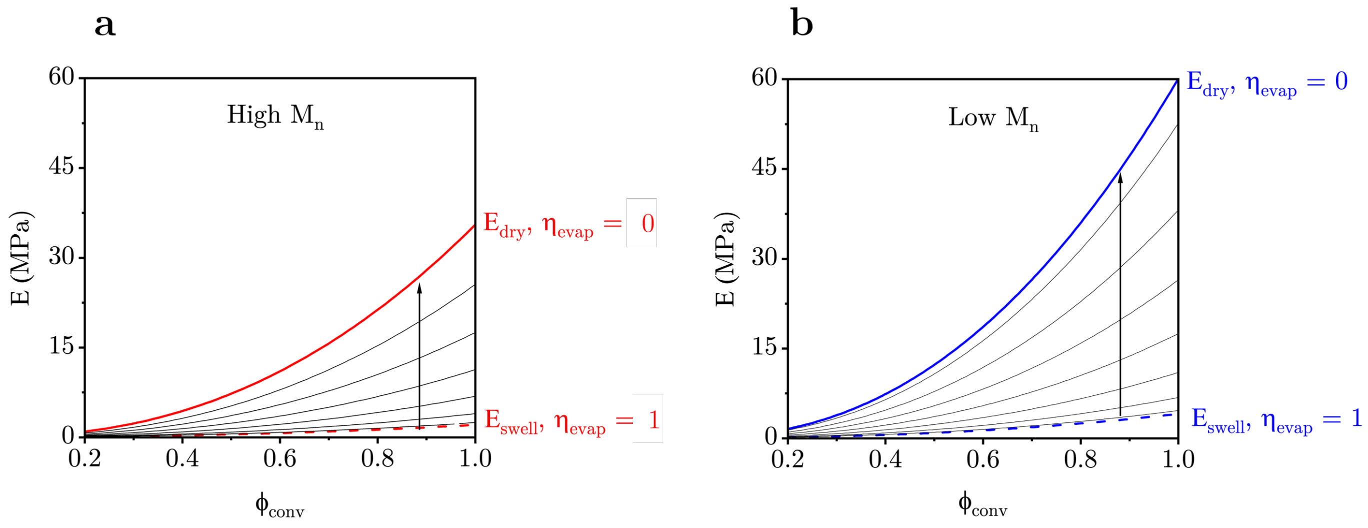

Figure S7 Young's modulus value for PEGDA used in simulations for (a) High molecular weight and (b) low molecular weight. The solid red, blue, and black lines correspond to low molecular weight PEGDA maximum, minimum, and variable evaporation ratio η_{evap}, in equal steps of 0.125, from 1 (swollen) to 1 (dry).

To quantify the curvature κ due to the solvent evaporation-diffusion in different networks, we incorporated ε and E resultant changes into Bernoulli's beam theory. κ can be defined as:

$$\kappa = \frac{\int_0^h E\varepsilon(z - z_N)dz}{\int_0^h E(z - z_N)^2 dz} \tag{S10}$$

with

$$z_N = \frac{\int_0^h Ezdz}{\int_0^h Edz} \tag{S11}$$

where z_N is the neutral axis. We compute κ and z_N by evaluating the integral numerically. To this end, we summarize all the data used in the simulation in the Table 2.

By using the values tabulated in Table S2, we obtain outputs for networks responses with different monomer M_n. We compared five parameters: $C(z,t)$, η_{evap}, E, ε, and σ (Fig. S8). While the solvent absorption capacity for both networks are different (see Fig. S3) $C(z,t)$ shows similar trends for both networks. Initially, $C(z,0)$ follows the equation of: $C(z,0) = C_0 \times \phi^{-n}$. As the drying process continues, the concentrations drop at $z = 0$ and $z = z_f$, due to the evaporation at boundaries. Over time, the solvent in the center part of the polymer diffuses to the boundaries as the concentration difference develops. This observation also reflects in η_{evap}. The major differences between networks with low and high monomer M_n are in E and ε. We set the initial Young's modulus (E_0, $\phi \geq \phi_c$) for both low and high monomer M_n to differ by 0.43 MPa, in line with the findings by Wang *et al.*[10] We differentiate ε response of low and high monomer M_n by adjusting the swelling, g, and drying constant, h, hence, the networks from low monomer M_n swell and shrink smaller compared to high monomer

Table S2 Parameters for dynamic curvature driven by evaporation-diffusion simulation

No.	Parameter	High M_n Value	Low M_n Value	Unit
1	Irradiation dose, d	15.2	45	mJ/cm^2
2	Critical monomer-to-polymer conversion, ϕ_C	0.2	0.2	
3	Conversion rate constant, K	0.029	0.01	cm^2/mJ
4	Optical attenuation coeffcient, μ	3.4	3.4	mm^{-1}
5	Critical swelling Young's modulus, $E_{C,swollen}$	2	4	MPa
6	Critical dry Young's modulus, $E_{C,dry}$	40	60	MPa
7	Initial Young's modulus, E_0	0.07	0.5	MPa
8	Power law conversion, b	2.3	2.3	
9	Power law solvent, a	2.3	2.3	
10	Swelling constant, g	1.8	2	
11	Drying constant, h	0.6	0.4	
12	Base diffusion constant, D_0	2×10^{-6}	1×10^{-6}	mm^2/s
13	Diffusion constant power law, α	1.5	1.5	
14	Evaporation coefficient, k_{evap}	0.0002	0.0002	mm/s
15	Evaporation coefficient power law, n	1/3	1/3	

M_n. Intuitively, this comes from the fact that low monomer M_n has a higher crosslinking density. To this end, we show significant differences in the internal stress distribution, σ, leading to different response in curvature κ.

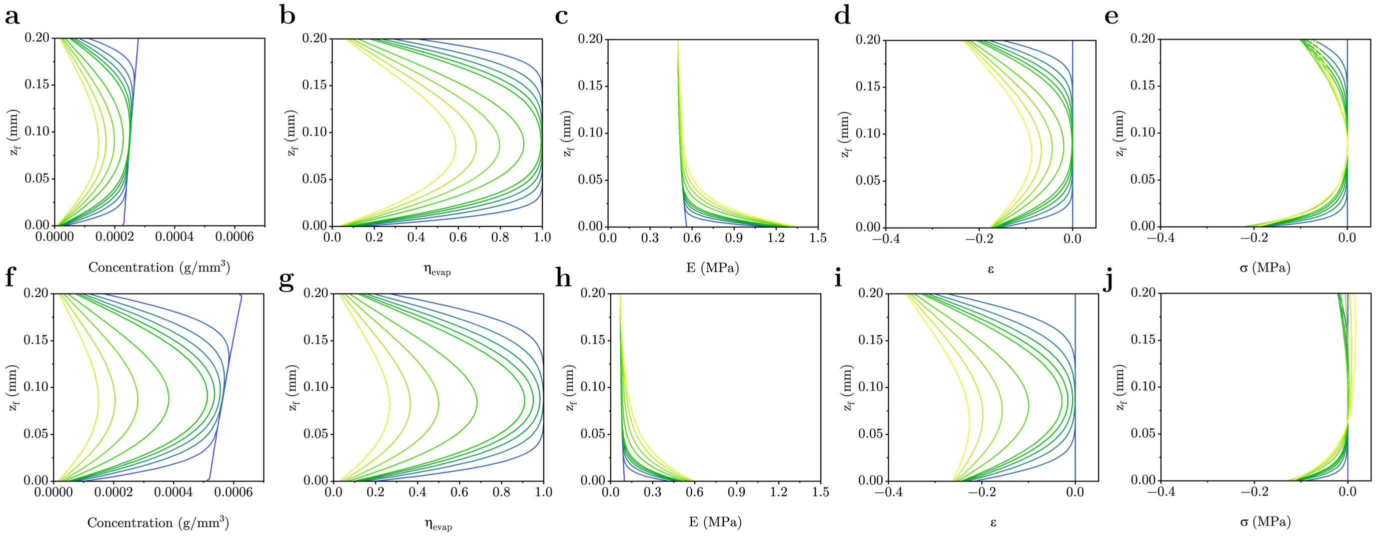

Figure S8 Simulation results of physical and mechanical parameters for networks with $z_f = 0.2$ mm, comprise of either high M_n((a) C(z,t), (b) η_{evap}, (c) E, (d) ε, (e) σ) or low M_n((f) C(z,t), (g) η_{evap}, (h) E, (i) ε, (j) σ). Each graph is simulated in the sequence of time 0, 20, 40, 60, 80, 100, 200, 300, 400, and 500 s.

S5 Additional data on spatiotemporal response of the networks

We next provide additional data for spatiotemporal responses of asymmetric networks with different $\bar{\mu}$ and monomer M_n. The networks is shaped in a starfish for easier imaging operations. However, at low $\bar{\mu}$, we use a rectangular beam shape for easier handling of swollen soft polymer. We initially fabricate the polymer networks and remove the excess unreacted monomer afterwards via pat drying. We then immerse the polymer in solvent for 3 minutes. Following solvent immersion, we remove excess solvent and expose the polymer in ambient air. The results are summarized below (Fig. S9).

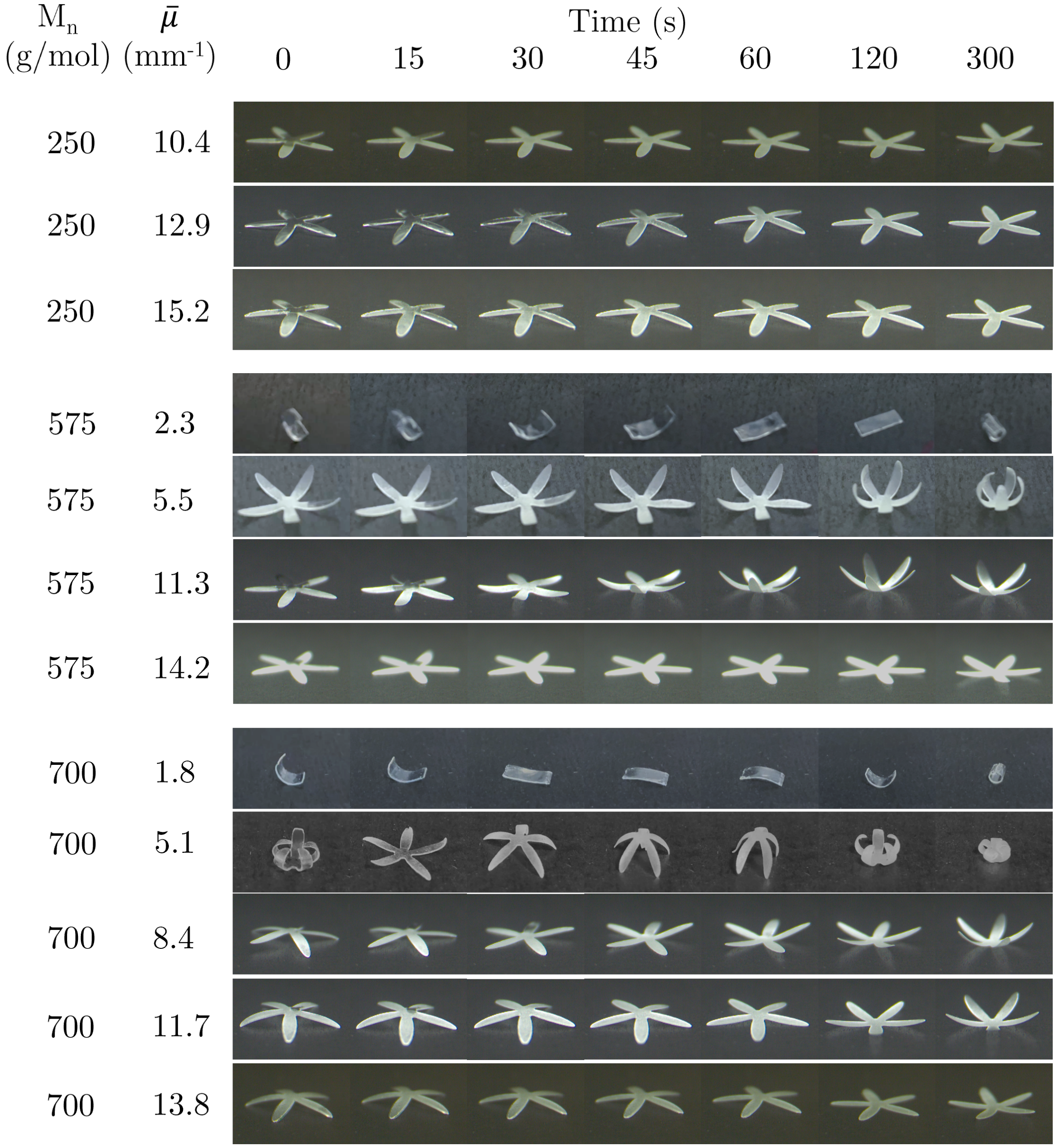

Figure S9 Spatiotemporal responses of asymmetric networks ($z_f = 0.2$ mm) with various monomer M_n and $\bar{\mu}$ during the ambient exposure after solvent immersion. The $\bar{\mu}$ values are modulated by adjusting PI concentration.

References

[1] A. Vitale, M. G. Hennessy, O. K. Matar and J. T. Cabral, *Advanced Materials*, 2015, **27**, 6118–6124.

[2] A. Vitale, M. G. Hennessy, O. K. Matar and J. T. Cabral, *Macromolecules*, 2015, **48**, 198–205.

[3] J. T. Cabral, S. D. Hudson, C. Harrison and J. F. Douglas, *Langmuir*, 2004, **20**, 10020–10029.

[4] J. T. Cabral and J. F. Douglas, *Polymer*, 2005, **46**, 4230–4241.

[5] M. G. Ridwan, Z. Ahmad, A. Vitale and J. T. Cabral, *Advanced Materials Interfaces*, 2026, e00879.

[6] H. Tokuyama, Y. Nakahata and T. Ban, *Journal of Membrane Science*, 2020, **595**, 117533.

[7] Z. Zhao, J. Wu, X. Mu, H. Chen, H. J. Qi and D. Fang, *Macromolecular Rapid Communications*, 2017, **38**, 1600625.

[8] Q. Zhang, X. Kuang, S. Weng, Z. Zhao, H. Chen, D. Fang and H. J. Qi, *ACS Applied Materials & Interfaces*, 2020, **12**, 17979–17987.

[9] Z. Li, Z. Liu, T. Y. Ng and P. Sharma, *Extreme Mechanics Letters*, 2020, **35**, 100617.

[10] J. Wang, X. Mu, D. Li, C. Yu, X. Cheng and N. Dai, *Advanced Engineering Materials*, 2019, **21**, 1801279.
